# Regulation of R1 Plasmid Transfer by H-NS, ArcA, TraJ, and DNA Sequence Elements

**DOI:** 10.3389/fmicb.2020.01254

**Published:** 2020-06-11

**Authors:** Karin Bischof, Doris Schiffer, Sarah Trunk, Thomas Höfler, Anja Hopfer, Gerald Rechberger, Günther Koraimann

**Affiliations:** Institute of Molecular Biosciences, University of Graz, Graz, Austria

**Keywords:** F-like plasmids, horizontal gene transfer, bacterial conjugation, type IV secretion, antibiotic resistance, gene silencing

## Abstract

In conjugative elements such as integrating conjugative elements (ICEs) or conjugative plasmids (CPs) transcription of DNA transfer genes is a prerequisite for cells to become transfer competent, i.e., capable of delivering plasmid DNA via bacterial conjugation into new host bacteria. In the large family of F-like plasmids belonging to the MobF_12_A group, transcription of DNA transfer genes is tightly controlled and dependent on the activation of a single promoter, designated P_Y_. Plasmid encoded TraJ and chromosomally encoded ArcA proteins are known activators, whereas the nucleoid associated protein heat-stable nucleoid structuring (H-NS) silences the P_Y_ promoter. To better understand the role of these proteins in P_Y_ promoter activation, we performed *in vitro* DNA binding studies using purified H-NS, ArcA, and TraJ_R__1_ (TraJ encoded by the conjugative resistance plasmid R1). All proteins could bind to R1P_Y_ DNA with high affinities; however, only ArcA was found to be highly sequence specific. DNase I footprinting studies revealed three H-NS binding sites, confirmed the binding site for ArcA, and suggested that TraJ contacts a dyad symmetry DNA sequence located between −51 and −38 in the R1P_Y_ promoter region. Moreover, TraJ_R__1_ and ArcA supplied together changed the H-NS specific protection pattern suggesting that these proteins are able to replace H-NS from R1P_Y_ regions proximal to the transcription start site. Our findings were corroborated by P_Y_-*lacZ* reporter fusions with a series of site specific R1P_Y_ promoter mutations. Sequential changes of some critical DNA bases in the TraJ binding site (*jbs*) from plasmid R1 to plasmid F led to a remarkable specificity switch: The P_Y_ promoter became activatable by F encoded TraJ whereas TraJ_R__1_ lost its activation function. The R1P_Y_ mutagenesis approach also confirmed the requirement for the host-encoded response-regulator ArcA and indicated that the sequence context, especially in the −35 region is critical for P_Y_ regulation and function.

## Introduction

Horizontal gene transfer by conjugation is ubiquitous among microorganisms belonging to the kingdoms of bacteria and archaea. If a transfer competent cell harboring a mobile genetic element contacts a recipient in a suitable environment, subsequent transfer of genetic information can decisively expand the metabolic, resistance, or virulence capabilities of the recipient bacterium. In canonical conjugation systems, single-stranded DNA is transported unidirectionally by means of a cell envelope-spanning multi-protein transport complex [termed type IV secretion system (T4SS)]. The process of DNA transfer through the T4SS is ATP dependent and coupled to DNA replication. Mechanistic and structural aspects are detailed in excellent recent reviews (Wong et al., 2012; Christie, 2016; Zechner et al., 2017; Waksman, 2019). The transported DNA is usually guided by at least one protein and derived from an autonomously replicating conjugative plasmid (CP) or from an integrating conjugative element (ICE). Importantly, additional cargo genes integrated into the mobile DNA element can rapidly spread throughout bacterial populations.

**FIGURE 1:**
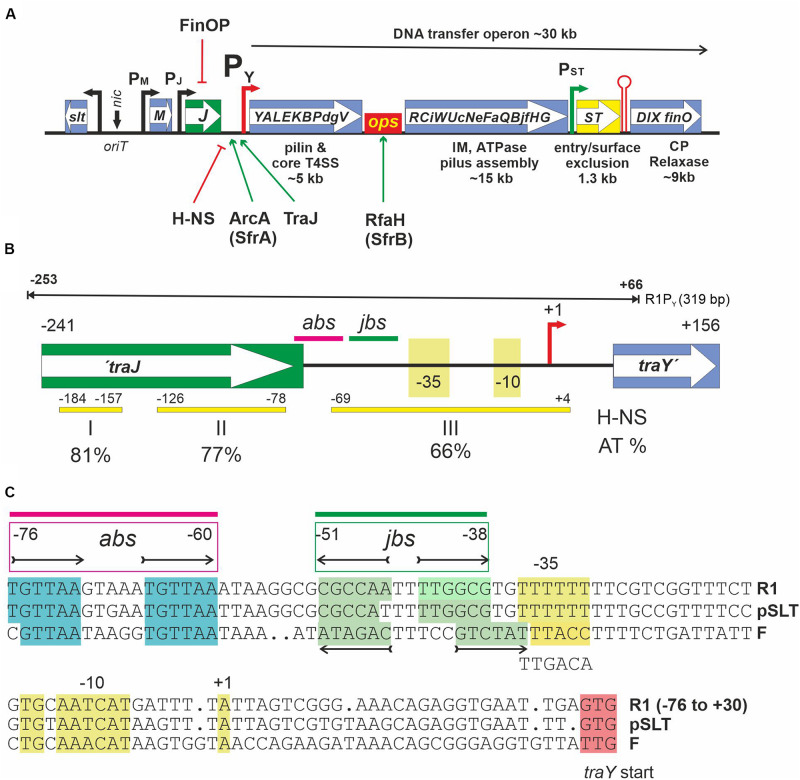
**(A)** Schematic representation of the DNA transfer region of F-like MOB_F__12_A plasmids. DNA transfer (*tra*) genes are indicated by capital letters inside the boxes. *oriT* is the origin of DNA transfer, representing a DNA region where several DNA binding proteins such as TraM, TraY, and TraI (the relaxase-helicase) bind. *nic* denotes the nick site which is recognized by the relaxase for the initiation of ssDNA transfer into recipients via a T4SS. Arrows indicate known promoters. P_Y_ is the only known promoter for transcription of the approximately 35 DNA transfer operon genes. It is regulated by the indicated host and plasmid encoded proteins. H-NS acts as a silencing factor whereas both ArcA (a.k.a. SfrA, host encoded) and TraJ (plasmid encoded) are needed for activation. Transcription of the complete operon additionally requires the host encoded anti-termination protein RfaH and its recognition site *ops* (operon polarity suppressor). Entry/surface exclusion genes *traS* and *traT* are constitutively transcribed in plasmid carrying cells. The transcriptional terminator (red hairpin) is functional only for transcripts initiating at promotor P_ST_. For a more in-depth discussion of DNA transfer genes and their regulation in MOB_F__12_A plasmids, the reader is referred to a recent review (Koraimann, 2018). **(B)** A schematic overview of the P_Y_ promoter region of the MOB_F__12_A prototype plasmid R1 from −241 to +156 relative to the transcription start site (+1, red arrow) of the *tra* operon is shown. This fragment is present in the promoter test plasmid pRSYZ4. Yellow bars with numbers indicating their location represent H-NS binding regions (I,II,III) as determined in this work. AT percentage of these regions is shown below. *abs*, binding site for ArcA; *jbs*, binding site for TraJ. These elements are also present on the 319 bp DNA fragment—termed R1P_Y_—used for EMSA and DNase I footprinting experiments (black line above). **(C)** Sequence representation of the P_Y_ promoter of MOB_F__12_A plasmids R1 (KY749247), pSLT (AE006471), and F (AP001918). To access a complete plasmid sequence in the NCBI nucleotide database (https://www.ncbi.nlm.nih.gov/nuccore), please use the accession number provided in parentheses. The non-template DNA strand is shown. *abs* and *jbs* sites as determined in this work are given above the sequences for the plasmid R1 P_Y_ promoter. The tandem repeat for ArcA binding [consensus: TGTTAA(N5)TGTTAA] is highlighted in cyan and the *jbs* inverted repeat sequences are highlighted in green. −10, extended −10 (TG), −35 as well as the first transcribed nucleotide (+1) are highlighted in yellow.

Within the *Enterobacteriaceae*, F-like plasmids are highly prevalent and have been found to be frequently associated with a variety of cargo genes such as antibiotic, biocide, and metal resistance genes. In addition, colicin and microcin genes as well as virulence and enterotoxin genes can be present, providing advantageous traits to their hosts (Koraimann, 2018). Due to the presence of a common backbone with DNA transfer, replication, toxin-antitoxin, and partitioning genes, this group of CPs represent highly successful mobile genetic elements that spread and persist in many enterobacterial species and importantly also in pathogenic strains of *Escherichia coli* (for a recent review, see Koraimann, 2018). Based on a classification scheme for CPs, F-like plasmids with the classical F plasmid or the antibiotic resistance plasmid R1 belong to the MOB_F__12_A group. They harbor conjugation genes that are phylogenetically related and share a similar arrangement (Fernandez-Lopez et al., 2016; Koraimann, 2018; Figure 1A). Nine subgroups have been defined within the MOB_F__12_A based on the amino acid sequence variability of the DNA transfer gene activator TraJ (Koraimann, 2018).

The ability to transport DNA from donors into recipient cells requires the expression of DNA transfer genes. A variety of regulatory mechanisms ensure that only a few cells in a genetically homogeneous population undergo this developmental process and become transfer competent. Thereby, at the population level, negative consequences of transfer gene expression are reduced without compromising horizontal gene transfer to novel hosts (Koraimann and Wagner, 2014; Stingl and Koraimann, 2017). In F-like plasmids DNA transfer genes are controlled through a network of regulatory elements ensuring that under optimal conditions only a few cells in a population—approximately 0.1 to 1%—proceed to transfer competence (Frost and Koraimann, 2010; Koraimann and Wagner, 2014). The key plasmid encoded activator that has to escape negative control is TraJ, a plasmid encoded protein. As schematically depicted in Figure 1A, TraJ and chromosomally encoded ArcA proteins are absolutely required to overcome heat-stable nucleoid structuring (H-NS) protein silencing and to activate transcription of DNA transfer genes from the main transfer operon promoter P_Y_ (Will and Frost, 2006; Wagner et al., 2013; Lu et al., 2018).

Here we characterize DNA binding characteristics of these proteins and focus on defining the binding sites in the P_Y_ promoter region of plasmid R1 (TraJ_R__1_), a conjugative resistance plasmid discovered 1963 in London in a clinical *Salmonella enterica* serovar Paratyphi B isolate (Datta and Kontomichalou, 1965). Originally named R_7268_, it was subsequently renamed R1 (Meynell and Datta, 1966). Only recently the complete sequence of this plasmid has been determined (Cox and Schildbach, 2017, accession: KY749247). In order to characterize DNA binding, purified H-NS, ArcA, and TraJ_R__1_ proteins were used in both electrophoretic mobility shift assays (EMSA) and DNase I footprinting experiments. EMSA revealed DNA binding by H-NS and TraJ with low sequence specificity, whereas ArcA binding to R1P_Y_ was highly sequence specific. In DNase I footprinting experiments, the TraJ_R__1_ binding site (*jbs*) in the P_Y_ promoter region was found to span the region from −51 to −38, immediately adjacent to the ArcA binding site (*abs*), located at −76 to −60 (see Figures 1B,C). Furthermore, TraJ and ArcA together were found to be capable of displacing the silencing factor H-NS from promoter proximal sites of the P_Y_ promoter region *in vitro*. In order to corroborate these findings, we mutated the proposed crucial sequence elements of the P_Y_ promoter and tested the mutants in a well-established reporter system. This approach confirmed the binding site for TraJ_R__1_ in the region −51 to −38 and revealed that specificity for binding by TraJ solely resides in this locus. Second, it confirmed the essential contribution of ArcA in P_Y_ promoter activation and finally demonstrated that the non-canonical −35 element is important for control and regulation of P_Y_.

## Materials and Methods

### Media, Growth Conditions, Bacterial Strains, Plasmids, and Oligonucleotides

LB medium (L^–1^: 10 g tryptone, 5 g yeast extract, 5 g NaCl) or 2xTY medium (per liter: 16 g tryptone, 10 g yeast extract, and 5 g NaCl) was used. For β-galactosidase assays, cells were grown in M9 minimal salt medium (Miller, 1972); L^–1^: 3 g KH_2_PO_4_, 12.8 g Na_2_HPO_4_ × 7 H_2_O, 1 g NH_4_Cl, 0,5 g NaCl, 3 g casaminoacids; 0.1 mg/mL thiamin; 0.2% glucose; 1 mM MgSO_4_. If appropriate, antibiotics such as 40 μg mL^–1^ kanamycin, 20 μg mL^–1^ chloramphenicol, or 50 μg mL^–1^ ampicillin were added. Unless otherwise indicated, cultures were grown aerated in a shaker-incubator at 180 r/min and 37°C. Cell densities (OD_600_) were measured in a Hitachi U5100 spectrophotometer. *E. coli* strains and plasmids are listed in Supplementary Table S1; oligonucleotides obtained from eurofins Genomics (Ebersberg, Germany) used in this study are listed in Supplementary Table S2.

### DNA Manipulations, Cloning, Sequencing, and Sequence Analyses

DNA manipulations were done using standard techniques (Sambrook et al., 1989) or according to the manufacturers’ recommendations. PCR reactions for cloning purposes were performed using Phusion High-Fidelity DNA Polymerase (New England BioLabs). Construction of expression plasmids for TraJ (pSD1002), TraJI_187__T_ (pSD1002_I__187__T_), and H-NS (pSThns) are detailed in the Supplementary Methods section. ArcA was overproduced from plasmid pETarcA-1 (Strohmaier et al., 1998). Mutations in the R1P_Y_-*lacZ* promoter test plasmid pRSYZ4 (Strohmaier et al., 1998; Wagner et al., 2013) were introduced according to a modified QuikChange protocol (Liu and Naismith, 2008; Laible and Boonrod, 2009). TraJ expressing plasmids in the R1P_Y_ promoter activation studies were the same as used previously (Wagner et al., 2013) except pJSlt2 containing the *traJ* gene from the *Salmonella enterica* plasmid pSLT. All resulting plasmids created in this study were verified by DNA sequencing using the Eurofins Genomics (Ebersberg, Germany) sequencing service. *In silico* construction of plasmids and sequence analysis was performed using SnapGene software.

### Protein Expression and Purification

ArcA protein containing an N-terminal His_6_ tag was purified using the method described and activated by phosphorylation before use in EMSA and DNase I footprintig assays (Strohmaier et al., 1998). TraJ and His_6_-H-NS were produced and purified as described in the Supplementary Material.

### DNA Fragments for EMSA and DNase I Footprinting Experiments

All DNA fragments were amplified by PCR using 5′-Cy5-labeled primers as indicated. Primers and their sequences are listed in Supplementary Table S2. R1P_Y_ DNA (319 bp) from −253 to +66 relative to the transcription start site of the *tra* operon containing the 3′ end of the *traJ* gene, the previously established ArcA-P binding site (Strohmaier et al., 1998), the predicted P_Y_ promoter (Strohmaier et al., 1998), and the 5′ end of the *traY* gene (Figure 1B) was amplified by PCR from *E. coli* harboring plasmid R1 using oligonucleotides PYTransfw, and PYTransrev1. For footprinting studies, only one of the two primers was 5′-Cy5-labeled. **GZ** DNA (261 bp) is a fragment generated from plasmid pGZ119EH DNA using oligonucleotides pTG-NF4-fw and pgzrev508. It roughly encompasses the *tac* promoter, the *lac* operator and the multiple cloning site of the plasmid. **FP_Y_** DNA (422 bp) was generated by PCR from *E. coli* XK1200 (pOX38-Km) and primers F_Py_fw and F_Py_rev. It contains DNA from −393 to +29 relative to the transcription start site of the F-plasmid *tra* operon. **Atu** DNA (395 bp) was generated by PCR from *Agrobacterium tumefaciens* C58 using primers GroEL_Atum_fw and GroEL_Atum_rev and covers a part of the *groEL* gene.

### EMSA

A Cy-5 labeled DNA fragment (0.5 nM) was mixed by pipetting on ice with varying concentrations of purified protein in band shift buffer (12 mM HEPES pH 7.9, 30 mM Tris-Cl pH 7.5, 60 mM KCl, 10% glycerol, 1.7 mM EDTA, 26 mM boric acid, 5 mM TCEP). Samples were placed in 0.2 mL PCR tubes; total reaction volume was 15 μL. Tubes were then transferred to a thermocycler and incubated at 30°C for 15 min. DNA and protein–DNA complexes were then electrophoretically separated on non-denaturing 6% polyacrylamide gels (PAGs; acrylamide:bisacrylamide 80:1, 2.5% glycerol, 0.07% APS, and 0.07% TEMED). Vertical electrophoresis was performed in Hoefer Mighty Small II chambers with TBE buffer (100 mM Tris base, 100 mM boric acid, 2.5 mM EDTA pH 8.3) with 20 mA at 4°C for 30–45 min. After disassembly of the electrophoresis unit, the gel was scanned using a Typhoon 9400 scanner (GE Healthcare). For image analysis and estimation of the dissociation constant, the Quantity One software (Bio-Rad) was used. Each band shift experiment was performed at least twice.

### DNase I Footprinting

For the DNase I footprint experiment, the same 319 bp R1P_Y_ DNA fragment as described for EMSA experiments was used with the exception that only one DNA strand was Cy5-labeled. Binding reaction mixtures (50 μL total volume) contained 50 mM Tris-Cl pH 7.2, 100 mM KCl, 10% glycerol, 30 ng of P_Y_ DNA, and varying concentrations of TraJ, ArcA-P, or H-NS. To allow protein–DNA complex formation, P_Y_ DNA and proteins were incubated for 20 min at 4°C and 2 min at 25°C. 50 μL of a 10 mM MgCl_2_, 5 mM CaCl_2_ solution were added and incubated for 1 min. To digest the DNA 0.1 U DNase I (1 U/μL) (Fermentas) were added directly to the protein-DNA mixes and incubated for 2 min. The reaction was terminated with 90 μL stop solution (20 mM EDTA, 200 mM NaCl, 1% SDS, 250 μg/mL herring sperm DNA). DNA was precipitated by adding 2.5 volumes of ethanol (96%) and incubation for 3 h at −70°C. Precipitates were sedimented by centrifugation (15,000 × *g*, 10 min, RT), washed with 70% ethanol, and centrifuged as before. After removal of the supernatant, the pellets were air-dried and resuspended in 10 μL formamide loading dye (Thermo Sequenase Primer Cycle Sequencing Kit, GE Healthcare), denatured for 10 min at 95°C, and separated on a 8% polyacrylamide sequencing gel (ReproGel^TM^, High resolution, GE Healthcare) in an Alf Express Sequencer (GE Healthcare). The sequencing reactions of the P_Y_ DNA fragment were done with the Thermo Sequenase fluorescent-labeled Primer Cycle Sequencing Kit (GE Healthcare) using 200 ng of DNA and Cy-5 labeled primer PYTransfw or PYTransrev1. Data were analyzed using the AlfWin Sequence Analyzer software (Amersham Pharmacia Biotech).

### R1P_Y_ Promoter Activation Tests

For these tests, *E. coli* MC4100 co-transformed with the P_Y_ promoter test plasmid pRSYZ4 (carrying a R1P_Y_-*lacZ* fusion) and *traJ* expressing plasmids (pJR1 or pJF or pJSLT2) or a vector control plasmid (pGZNSO2) were assayed for β-galactosidase activity. Promoter activation tests were similarly performed with derivatives of pRSYZ4 created by site specific mutagenesis. β-galactosidase assays were performed essentially as described previously (Wagner et al., 2013) and according to an Open Wetware protocol^1^ (last accessed 28/02/2020). Details of the procedure can be found in Supplementary Material.

## Results

### Binding of H-NS ArcA, and TraJ to R1P_Y_ DNA

**FIGURE 2:**
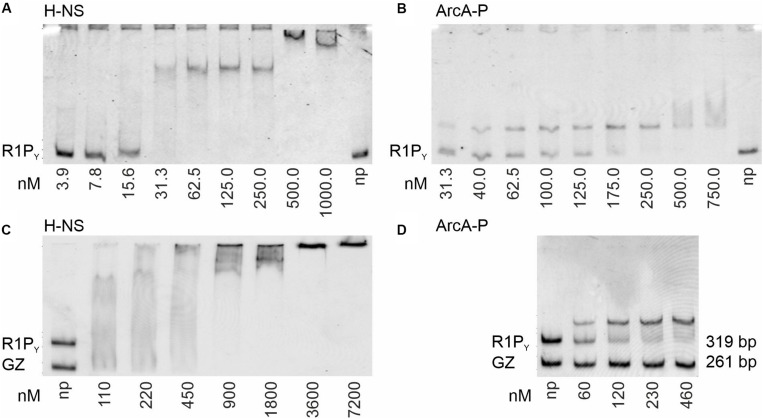
Binding of H-NS and ArcA-P (phosphorylated ArcA) to R1P_Y_ (319 bp, 62% AT, see Figure 1B) and control (GZ, 261 bp, 49% AT) DNA fragments. Electrophoretic mobility shift assays (EMSAs) were performed with the indicated fluorescently Cy5-labeled ds DNA fragments (0.5 nM) and purified proteins using the indicated protein concentrations (np: no protein present). Formed DNA–protein complexes were electrophoretically separated using non-denaturing polyacrylamide gels (PAGs) from free DNA and visualized by fluorescence imaging. Free DNA fragments are indicated at the bottom of the gels. Complexes appear as distinct bands, smear, or are retained in the gel slots. To increase perceptual contrast, images were inverted (gray fluorescence signal on a white background). **(A)** EMSA with purified H-NS protein and R1P_Y_ DNA. High affinity binding in with an apparent dissociation constant (*K*_*d*_) of about 15 nM can be observed. **(B)** EMSA with ArcA-P protein demonstrates binding of ArcA-P to R1P_Y_ with K_*d*_ of 60 nM. **(C)** H-NS binds to both R1P_Y_ and GZ DNA. **(D)** ArcA-P is highly specific and only binds to R1P_Y_ which contains the *abs* tandem repeat sequence as depicted in Figure 1C.

In a previous study, we could show that H-NS is involved in silencing *tra* gene expression of plasmid R1 *in vivo* (Wagner et al., 2013). We therefore set up experiments to investigate direct H-NS binding to a fluorescently labeled DNA fragment containing the R1 P_Y_ promoter (R1P_Y_ DNA, 319 bp, 62% AT) as shown in Figure 1B. This DNA fragment was incubated with increasing concentrations of purified H-NS protein and the resulting DNA–protein complexes were separated electrophoretically on a non-denaturing PAG. As can be seen in Figure 2A, H-NS bound to R1P_Y_ with high affinity producing a distinct complex visible at protein concentrations between 30 and 250 nM H-NS. Higher H-NS concentrations shifted the DNA to the top of the gel. We estimated an apparent dissociation constant *K*_*d*_ of 15 nM. However, as is clearly obvious from Figure 2C, H-NS also bound to a control DNA fragment (GZ DNA, 261 bp, 49% AT). For a direct comparison in the DNA binding behavior, we performed binding studies using the same DNA fragments with phosphorylated ArcA protein (ArcA-P). The results are shown in Figures 2B,D. Binding of ArcA-P to R1P_Y_ with apparent *K*_*d*_ of 60 nM is highly specific. ArcA-P only bound to R1P_Y_ containing the *abs* site shown in Figure 1C but not to the GZ fragment which does not contain a binding motif for ArcA.

**FIGURE 3:**
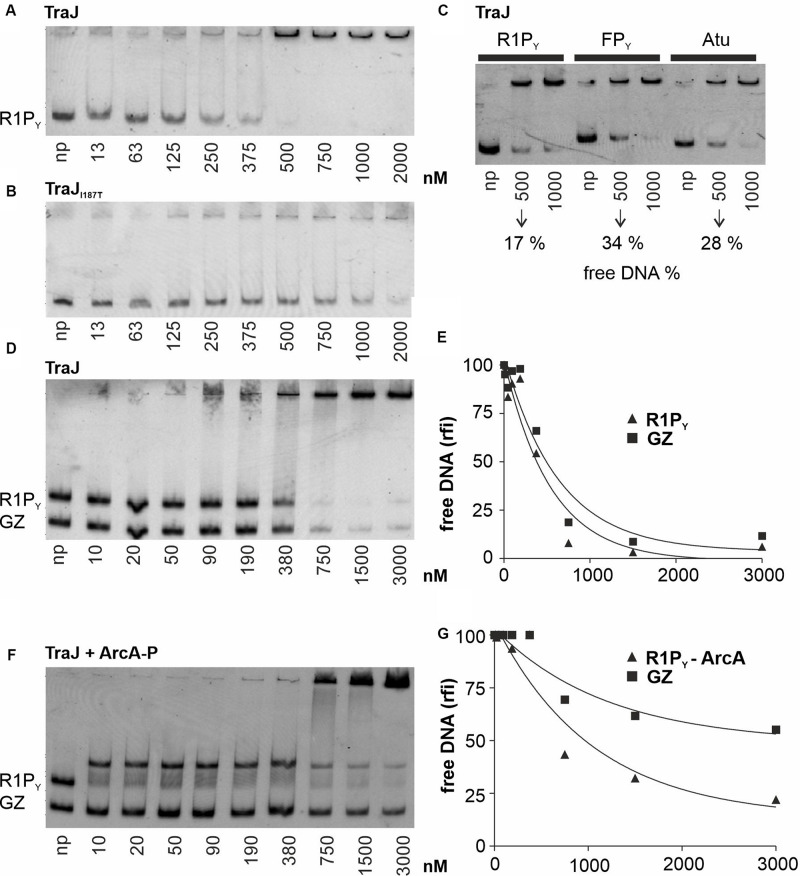
EMSAs with various proteins and DNA fragments to characterize TraJ_R__1_ binding to DNA. Binding studies were analyzed as described in Figure 2. np, no protein added. **(A)** TraJ_R__1_ binds to R1P_Y_ DNA. No distinct DNA–protein complexes can be seen. The *K*_*d*_ is estimated from this EMSA is 250 nM. At concentrations > 500 nM R1P_Y_ DNA is shifted to the gel slot. **(B)** An activation deficient TraJ_R__1_ mutant (I187T) displays a considerably weaker DNA binding activity with an apparent *K*_*d*_ of 750 nM. **(C)** Comparison of TraJ_R__1_ binding to different DNA fragments. TraJ_R__1_ binds with higher affinity to R1P_Y_ (319 bp) when compared to FP_Y_ (422 bp) or a DNA fragment from *Agrobacterium tumefaciens* (Atu, 395 bp). **(D)** Simultaneous binding of TraJ_R__1_ to R1P_Y_ and a control fragment (GZ). TraJ_R__1_ binds with higher affinity to R1P_Y_ when compared to a control DNA. **(E)** The percentage of free R1P_Y_ DNA and GZ DNA from **D** was calculated and graphically represented relative to the DNA fluorescence of the respective fragment without protein. **(F)** Pre-incubation of the DNA fragments (R1P_Y_ and GZ) with 250 nM ArcA-P increases TraJ_R__1_ preference for R1P_Y_ DNA. **(G)** The percentage of R1P_Y_ DNA bound to ArcA-P or free GZ DNA from **F** was calculated and graphically represented relative to the DNA fluorescence of the respective fragment without addition of TraJ_R__1_. The difference between TraJ_R__1_ binding to R1P_Y_-ArcA-P versus binding to GZ when compared to **E** is clearly increased. rfi: relative fluorescence intensity.

In a second series of band shift experiments (Figure 3), we wished to investigate the DNA binding properties of TraJ_R__1_ which has been identified as an essential activator of the P_Y_ promoter and *tra* operon transcription of plasmid R1 (Wagner et al., 2013). As can be seen in Figure 3A, purified TraJ_R__1_ bound to R1P_Y_ DNA; however, no distinct DNA–protein complexes appeared except that at concentrations > 500 nM TraJ_R__1_ R1P_Y_ DNA is shifted to the top of the gel. The apparent *K*_*d*_ was estimated to be 250 nM for binding of TraJ_R__1_ to R1P_Y_ DNA. Importantly, the only protein found by mass spectroscopy in the top shifted band was TraJ_R__1_ (data not shown). Thus, we exclude the presence of contaminating DNA binding proteins in our TraJ protein preparations. We previously identified a loss-of-function mutant of TraJ_R__1_ (I187T); furthermore, ChIP experiments suggested that this mutant had severely disturbed the DNA binding capability (Wagner et al., 2013). Consistent with these findings, purified TraJ_R__1_ (I187T) displayed a considerably weaker DNA binding activity with an apparent *K*_*d*_ of 750 nM (Figure 3B). To investigate the specificity of TraJ_R__1_ DNA binding, we performed several DNA binding experiments. As can be seen in Figure 3C, TraJ_R__1_ also bound to FP_Y_ and Atu DNA fragments which do not contain the *jbs* recognition sequence (Figure 1C). However, quantitation of the remaining free DNA in relation to total DNA at 500 nM TraJ_R__1_ revealed only 17% in case of R1P_Y_, whereas there were 34 or 28% in case of FP_Y_ or Atu fragments, respectively. This result suggested that TraJ_R__1_ bound with a higher affinity to R1P_Y_ (319 bp) when compared to FP_Y_ (422 bp) or a DNA fragment from *A. tumefaciens* (Atu, 395 bp). In another approach, TraJ_R__1_ binding was investigated using two different DNA fragments (R1P_Y_ and GZ) simultaneously. Although not a strikingly clear difference (compare ArcA-P binding to the same fragments, Figure 2D), there is a slight preference for the R1P_Y_ fragment (Figures 3D,E). We finally found that pre-incubation of the DNA fragments (R1P_Y_ and GZ) with 250 nM ArcA-P increased TraJ_R__1_ preference for R1P_Y_ DNA (Figures 3F,G). Taken together, these results suggest that TraJ_R__1_ has a DNA binding activity which is stronger when the cognate *jbs* site is present and that ArcA-P binding could direct TraJ_R__1_ toward the DNA containing *jbs* next to *abs*.

### H-NS and TraJ_R__1_ Binding Sites in the P_Y_ Promoter Region of Plasmid R1

**FIGURE 4:**
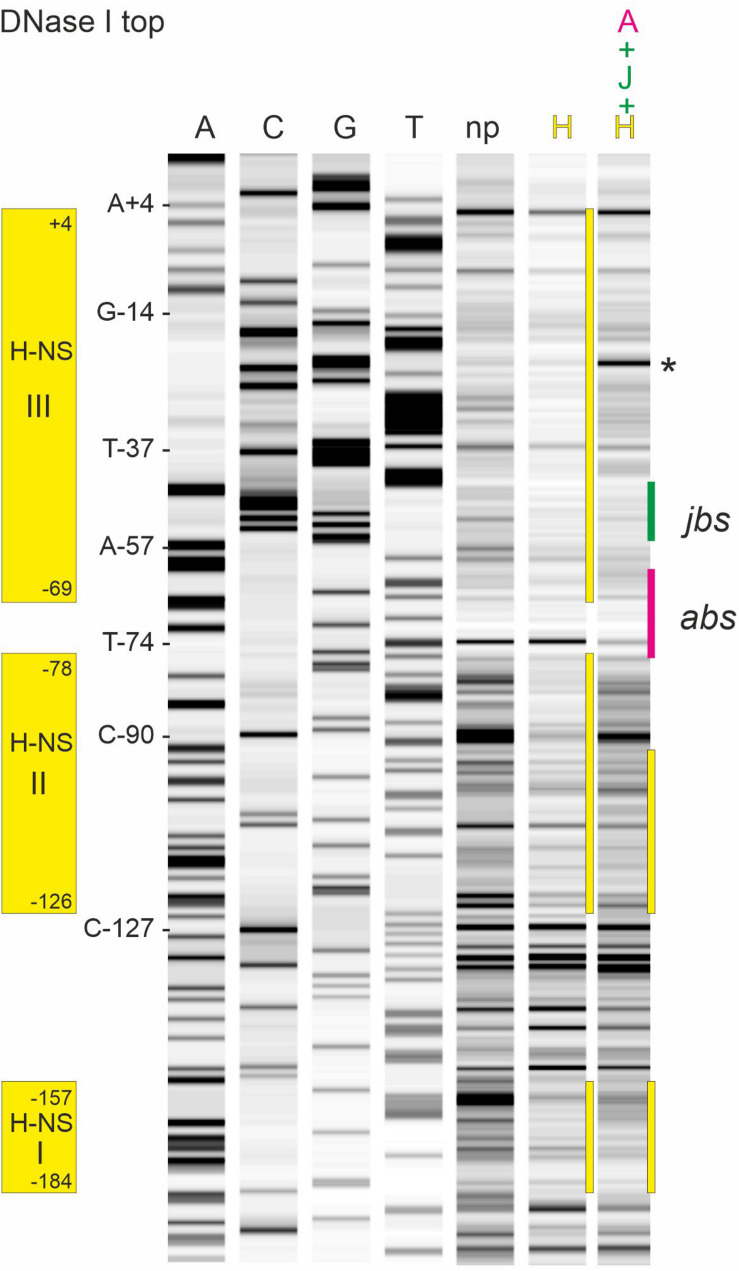
DNase I footprinting of the R1P_Y_ dsDNA fragment with the non-template strand fluorescently labeled reveals binding sites for H-NS, ArcA-P, and TraJ_R__1_ in the R1P_Y_ promoter region. Lanes A, C, G, and T are products of sequencing reactions with some of the bases indicated on the left side of lane A. np, DNA fragments obtained with DNase I digestion. H, DNA fragments obtained with 0.2 μM H-NS before DNase I digestion. Three distinct regions with reduced band intensities are indicated by yellow bars. H-NS protected regions are indicated on the left side and correspond to three AT rich regions as shown in Figure 1B. Last lane shows DNase I fragments obtained in the presence of ArcA-P (0.4 μM), TraJ_R__1_ (5 μM), and H-NS (0.2 μM). The resulting change in the banding pattern suggests a replacement of H-NS from binding site III and partially from binding site II. Note the appearance of an additional prominent band between −35 and −10 regions in the P_Y_ promoter (indicated by an asterisk). *abs*, binding site for ArcA-P; *jbs*, binding site for TraJ_R__1_.

Binding of ArcA-P to the R1 P_Y_ promoter region has been determined earlier by DNase I footprinting (Strohmaier et al., 1998) and clearly demonstrated that ArcA-P can bind to the *abs* site indicated in Figure 1C. To determine the binding sites for H-NS and TraJ_R__1_, we again performed DNase I protection assays using the 319 bp R1P_Y_ DNA fragment either fluorescently labeled on the top (non-coding) or bottom (coding) strand. The result of such an experiment is shown in Figure 4 where the binding regions for H-NS appear as three distinct regions with reduced band intensities when compared to the control DNase I digest without protein. These regions are labeled H-NS I (−184 to −157, 81% AT), H-NS II (−126 to −78, 77% AT), and H-NS III (−69 to + 4, 66% AT). The banding pattern changes when proteins ArcA-P and TraJ_R__1_ are additionally present (lane A + J + H in Figure 4), suggesting a replacement of H-NS by ArcA-P and TraJ_R__1_. The binding sites for ArcA-P and TraJ_R__1_ are indicated in Figure 4 and correspond to *abs* and *jbs* sequence motifs shown in Figure 1C.

**FIGURE 5:**
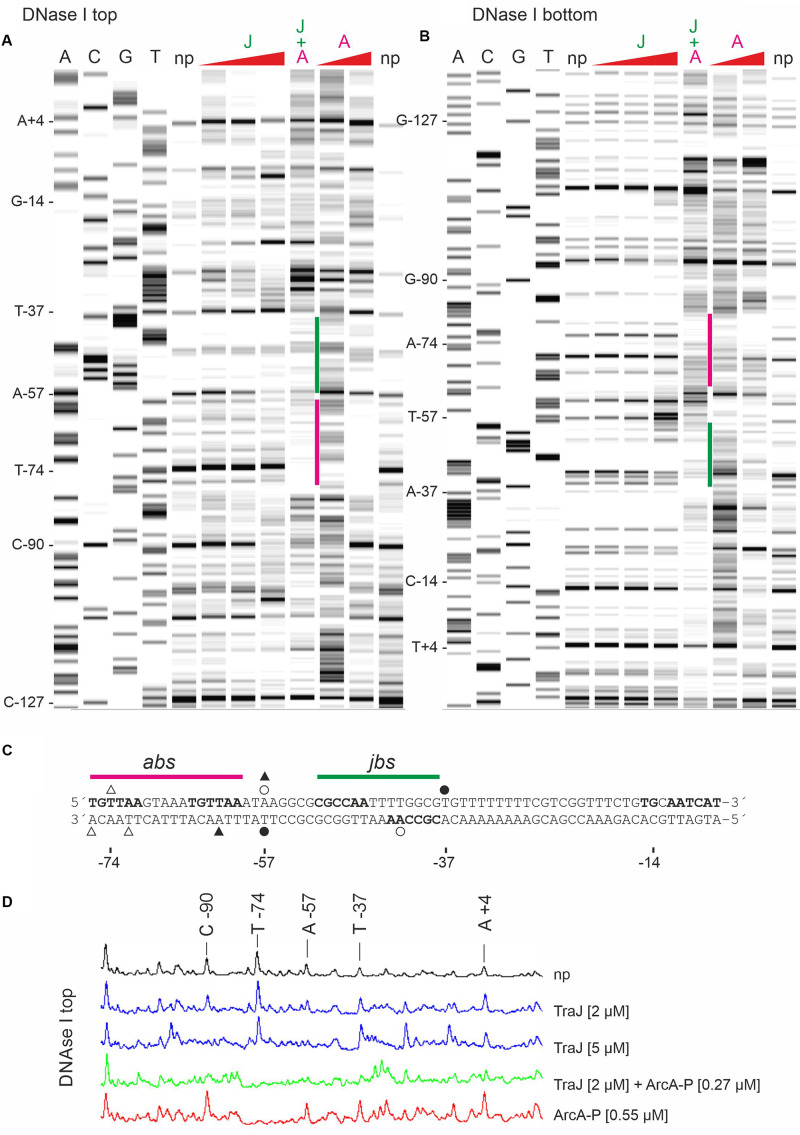
DNase I protection pattern by TraJ_R__1_ and ArcA-P obtained on R1P_Y_ DNA. R1P_Y_ DNA fluorescently 5′-labeled on the non-template **(A)** or the template **(B)** strand was incubated with proteins as indicated and subsequently digested with DNase I. The resulting DNA fragments were separated on a denaturing PAG. A gel-like image of the detected fluorescence signals is shown in **A,B**. Lanes A, C, G, and T are products of sequencing reactions. For orientation, some bases are indicated on the left side of lane A, respectively. Proteins were added before DNase I digestion as follows: J: TraJ_R__1_ 0.5, 2, or 5 μM; A: ArcA-P 0.27 or 0.55 μM. J + A: TraJR1 + ArcA-P, 2 and 0.27 μM, respectively. np, no protein added before DNase I digestion. A: TraJ_R__1_ protects from DNase I cleavage at positions A-57 and C-90. ArcA-P protects T-74. Positions A-57 and T-74 are consistent with the proposed *jbs* and *abs* recognition sites as shown in **C**, respectively. Addition of both proteins produces a protection pattern indicating simultaneous DNA binding of TraJ_R__1_ (indicated by a green bar) and ArcA-P (magenta bar). **(B)** TraJ_R__1_ protects from DNase I cleavage at position A-42. ArcA-P protects T-72 and A-76. These positions are consistent with the proposed *jbs* and *abs* regions as shown in **C**, respectively. Addition of both proteins produces a protection pattern indicating simultaneous DNA binding. **(C)** DNA sequences of non-template (top) and template (bottom) DNA strands from the R1P_Y_ promoter region (−76 to −7). The proposed *abs* (−76 to −60) and *jbs* (−51 to −38) sites are indicated above the sequence. Open triangles indicate bases protected by ArcA-P and filled triangles represent ArcA-P induced DNase I cleavage sites. Open circles indicate bases protected by TraJR1 and filled circles represent TraJ_R__1_ induced DNase I cleavage sites. + 1: transcription start site as determined by reverse transcription of *tra* mRNA, −10: −10 region of the P_Y_ promoter, TG: extended −10 motif. **(D)** Curve view of DNase I footprinting experiment shown in **A**. Proteins were added as indicated. np, no protein added before DNase I treatment.

To better define the binding site for TraJ_R__1_ further DNase I footprinting experiments were performed with increasing TraJ_R__1_ concentrations, ArcA-P alone and TraJ_R__1_ together with ArcA-P. As shown in Figures 5A,D (top strand labeled) TraJ_R__1_ protected from DNase I cleavage at positions A-57 and C-90. Protection of C-90 by TraJ_R__1_ indicates a secondary binding site in R1P_Y_ which is located outside the proposed *jbs* recognition site. This can be attributed to the sequence independent DNA binding activity of TraJ_R__1_ which was also evident in the band shift experiments. ArcA-P protected T-74. Positions A-57 and T-74 are consistent with the proposed *jbs* and *abs* recognition sites, respectively. Addition of both proteins produced a protection pattern indicating simultaneous DNA binding of TraJ_R__1_ and ArcA-P. On the bottom strand TraJ_R__1_ protected from DNase I cleavage at position A-42. ArcA-P protected at T-72 and A-76 (Figures 5B,C). Again, these positions are consistent with the proposed *jbs* and *abs* motifs. From these results, we deduce that the recognition site for TraJ_R__1_ (*jbs*) in the R1P_Y_ promoter extends from −51 to −38 and includes the imperfect inverted repeat sequence CGCCAATTTTGGCG with two non-palindromic bases (TT) in the center. Incubation of R1P_Y_ DNA with ArcA-P alone resulted in the expected protection of the direct repeats TGTTAAGTAAATGTTAA from −76 to −60. Binding of ArcA-P to this site has been shown by our group previously (Strohmaier et al., 1998). This sequence closely resembles the proposed recognition matrix for ArcA-P in *E. coli* (Liu and De Wulf, 2004; Park et al., 2013). In addition, the sequence motif is highly conserved in P_Y_ promoter sequences of F-like plasmids.

### P_Y_ Promoter Sequence Requirements for TraJ Activation

**FIGURE 6:**
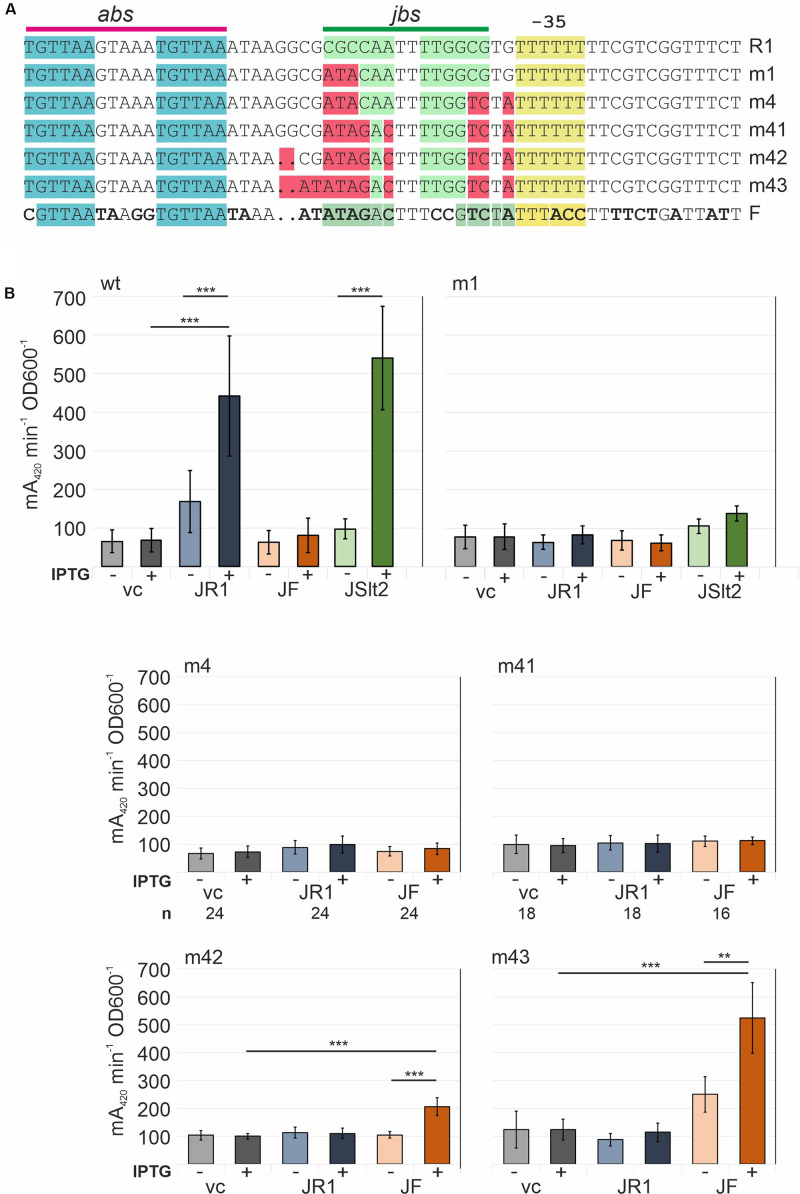
Determination of important sequence elements in the R1P_Y_ promoter for activation by TraJ_R__1_. Successively introduced mutations switch recognition specificity to TraJ_F_. **(A)** Sequences showing sequentially created site specific mutations in R1P_Y_. Top line shows the R1P_Y_ wt sequence with the *abs* and *jbs* sites indicated above. Bottom line shows FP_Y_ wt sequence with bases that differ from R1P_Y_ in bold letters. **(B)** β-Galactosidase assays with bacterial cells harboring a R1P_Y_-*lacZ* promoter-test plasmid and a second compatible plasmid IPTG-inducible for expression of *traJ* from plasmids R1 (JR1), F (JF), or pSLT (JSlt2); vc, vector control (no *traJ*). IPTG (inductor): +(added), −(not added). Mean values and standard deviations were calculated from at least three independently carried out experiments with two technical replicates for each experiment. *P*-values (paired *t*-test): **P < 0.01; ***P < 0.001.

The *in vitro* results demonstrated that TraJ_R__1_ can bind to DNA. Based on the above described preference for *jbs* containing DNA and the DNAse I footprinting data, we concluded that TraJ_R__1_ specifically recognizes an inverted repeat sequence in the P_Y_ promoter including bases from −51 to −38 in the R1P_Y_ promoter region. This is consistent with previous results of ChIP experiments where we could demonstrate specific *in vivo* binding of TraJ_R__1_ to the R1P_Y_ DNA (Wagner et al., 2013). We also noted that sequences in the P_Y_ promoter region between different subclasses of F-like plasmids differ as do the cognate TraJ proteins (Wagner et al., 2013; Koraimann, 2018). To verify the *jbs* recognition sequence and to demonstrate subclass-specific activation of the P_Y_ promoter of F-like plasmids, we systematically mutagenized the *jbs* site in the R1P_Y_ promoter and tested its activation by TraJ proteins from plasmid R1, pSLT, or F. The mutations shown in Figure 6A were introduced in the promoter test plasmid pRSYZ4 in which the R1P_Y_ promoter is fused to the *lacZ* reporter gene. β-Galactosidases assays were performed to evaluate basal promoter activity (with a vector control plasmid) and its activation by the above mentioned TraJ proteins. As can be seen in Figure 6B, there was a very low basal expression level from the R1P_Y_ promoter which could be induced by both TraJ_R__1_ and TraJ_pSLT_ but not by TraJ_F_. In the fully stimulated promoter expression was about 6.5-fold to 8-fold higher than the basal expression. The mutation of three bases (m1) in *jbs* did not change the basal expression level but completely abolished activation by TraJ_R__1_ showing that these bases in *jbs* are absolutely essential for recognition by TraJ_R__1_. There was no activation by TraJ_F_. Further sequential mutations toward the F *jbs* did not change this phenotype dramatically (m4, m41). However, finally, mutants with an almost completely changed *jbs* sequence (m42, m43) displayed a specificity switch. The P_Y_ promoter in these mutants was activated by TraJ_F_ and not by TraJ_R__1_. We also observed an approximately twofold increase in the basal P_Y_ promoter activity in mutant m43 which could be activated by TraJ_F_ fourfold (Figure 6).

**FIGURE 7:**
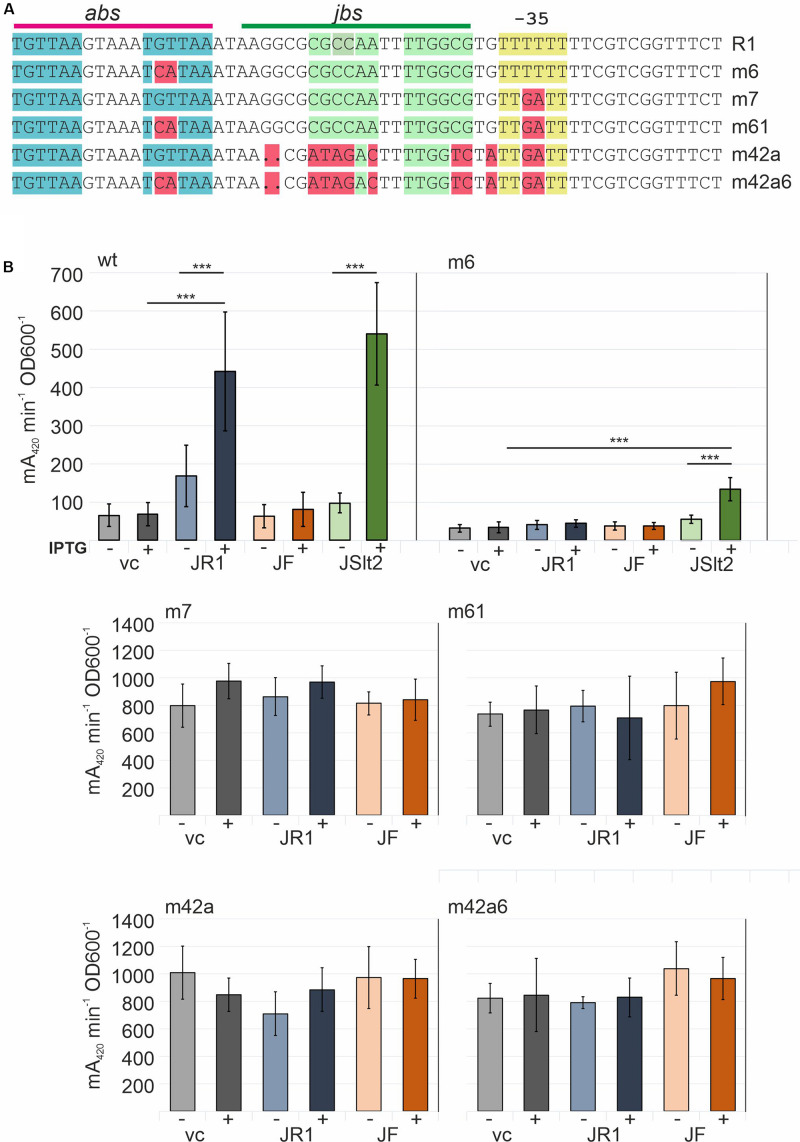
Determination of the role of *abs* and the non-canonical −35 region in the R1P_Y_ promoter. **(A)** Top line shows the R1P_Y_ wt sequence with the *abs* and *jbs* sites indicated above. Mutations tested in β-galactosidase assays are shown in the R1P_Y_ sequence context and highlighted in red. **(B)** β-Galactosidase assays with bacterial cells harboring a R1P_Y_-*lacZ* promoter-test plasmid and a second compatible plasmid IPTG-inducible for expression of *traJ* from plasmids R1 (JR1), F (JF), or pSLT (JSlt2). vc: vector control (no *traJ*). IPTG (inductor): +(added), -(not added). Mean values and standard deviations were calculated from at least three independently carried out experiments with two technical replicates for each experiment. *P*-values (paired *t*-test): ***P < 0.001.

Another series of mutations was introduced into the R1P_Y_ promoter to investigate the effects of exchanges in *abs* and the −35 region (Figure 7). In the m6 mutation in which the second TGTTAA ArcA recognition motif in *abs* is changed to TCATAA, we observed a reduced basal expression level (approximately 50% of the wt promoter) and a complete loss of its activation by TraJ_R__1_. This is consistent with earlier R1P_Y_ promoter activity determinations using *arcA* mutant strains (Wagner et al., 2013). Interestingly, there is some promoter activation by TraJ_pSLT_ which is probably due to a somewhat higher expression of this protein variant (data not shown). The −35-region mutant (TTTTTT to TTGATT) unexpectedly led to a complete different of the R1_PY_ promoter behavior. First, the basal expression level was higher than from the wt promoter in the fully induced state, second, there was no observable induction by TraJ, and third, there was no more dependence on ArcA since the abs site mutation had no effect on the high expression level. Thus, by introducing just two bases making the −35 sequence more similar to the canonical −35 recognition sequence (TTGACA) which is contacted by region 4 of the σ^70^ subunit of the RNA polymerase converted the R1P_Y_ promoter into a constitutive and completely deregulated promoter.

## Discussion

Expression of the *tra* operon is an absolute requirement for bacteria carrying CPs to develop transfer competence. Only transfer competent cells which usually only represent a minor subpopulation of plasmid harboring cells can actively transfer DNA into recipients and thereby spread genes horizontally in bacterial populations. Intricate genetic regulatory networks ensure that transfer genes are expressed only under optimal conditions (Frost and Koraimann, 2010; Koraimann and Wagner, 2014; Stingl and Koraimann, 2017). Here, we investigated the molecular basis of *tra* gene activation of the classical antibiotic resistance plasmid R1 which belongs to a large family of F-like plasmids, also categorized as MOB_F__12_A (Fernandez-Lopez et al., 2017). The performed experiments are based on a previous study where we genetically characterized the contribution of *hns*, *arcA*, and *traJ* on silencing and activating DNA transfer and type IV secretion genes of plasmid R1 (Wagner et al., 2013). H-NS binding to the R1P_Y_ promoter DNA could be directly shown by EMSA and DNase I footprinting experiments. As expected, DNA binding by H-NS was not specific for the R1P_Y_ promoter but also bound to a control DNA fragment in the EMSA with a lower AT content. However, the observed apparent *K*_*d*_ with the R1P_Y_ fragment was very low (15 nM) and comparable to DNA fragments containing nucleation sites for H-NS such as *csgD* or *proU* promoters (Bouffartigues et al., 2007; Gulvady et al., 2018). In the DNase I protection experiment, we identified three AT binding regions in the R1P_Y_ promoter region (H-NS I, H-NS II, and H-NS III). Due to their high AT content of 81 and 77%, the first two could serve as nucleation sites since this high AT content represents the documented preferred composition of H-NS targets *in vivo* (Navarre et al., 2006). Our results are fully consistent with the proposed silencing role of *hns* in *tra* gene expression of plasmid R1 (Wagner et al., 2013).

Counter-silencing functions of the known activators of *tra* gene expression ArcA (the response regulator of the ArcAB two component system) and TraJ, requires displacement of H-NS from at least the H-NS III binding region which could facilitate RNA polymerase access to −35 and −10 regions of the P_Y_ promoter. *In vitro* binding of ArcA-P and *in vivo* activation functions have been established earlier suggesting that these proteins could help clearing the promoter from H-NS and recruiting RNA polymerase (Strohmaier et al., 1998; Wagner et al., 2013). The EMSA results confirmed specific ArcA-P binding the R1P_Y_ promoter, in addition TraJ_R__1_ bound to DNA but only with a slight preference for R1P_Y_ DNA. DNase I footprinting analysis nevertheless revealed a protection pattern for TraJ_R__1_ that is consistent with the proposed *jbs* site with the inverted repeat sequence located between −51 and −38 and confirmed ArcA-P binding immediately next to TraJ_R__1_ to two direct repeats from −76 to −60. The TGTTAA hexamer represents the core of the ArcA sequence motif with the highest conservation. In most *E. coli* ArcA regulated promoters, two or three direct repeats of these ArcA boxes are found (Park et al., 2013). Since the simultaneous addition of both proteins can change the protection pattern of H-NS in a way that suggests replacement of H-NS by ArcA and TraJ from binding site III and partially from binding site II (Figure 4), we propose that such a mechanism is responsible for the observed *in vivo* activation of R1P_Y_ by ArcA and TraJ. Phosphorylated ArcA may be responsible for a first counter-silencing activity and opening the P_Y_ promoter for TraJ activator binding in a second step. It has similarly been shown that the SsrB response regulator of *S. enterica* can replace H-NS from type III effector gene promoters in SPI-2 required for intracellular growth and maintenance of this pathogen (Walthers et al., 2011). A cooperative activity of TraJ_F_ and ArcA has also recently been proposed to mediate activation of the FP_Y_ promoter which is also silenced by H-NS (Will and Frost, 2006; Rodriguez-Maillard et al., 2010; Lu et al., 2018). The results of our promoter mutagenesis studies fully supported the idea that counter-silencing in the R1P_Y_ promoter requires ArcA since when only two bases in the second ArcA box hexamer were exchanged (from TGTTAA to T**CA**TAA), together with a lower basal activity, induction of the R1P_Y_ promoter was virtually abolished. On the other hand, silencing required a non-canonical −35 hexamer (TTTTTT) which when mutated to TT**GA**TT completely disrupted silencing an converted the mutant promoter into a constitutive promoter independent of both ArcA and TraJ. We envision that this mutation disrupts binding of H-NS in H-NS III of the R1P_Y_ promoter and at the same time allows binding of the RNA polymerase holoenzyme (with σ^70^) to productively initiate transcription from R1 P_Y_.

Although the role of the plasmid encoded activator protein TraJ is well established, the exact mechanism how TraJ acts at the molecular level is still unclear. Whereas the EMSA experiments showed DNA binding with an apparent dissociation constant *K*_*d*_ of about 250 nM only revealed a weak preference for R1P_Y_ fragment which was somewhat enhanced by ArcA. Clearly, as shown in the DNase I footprints, TraJ was able to bind next to ArcA in the R1P_Y_ promoter. However, there was also a prominent protected band outside the TraJ recognition motif *jbs* suggesting binding to DNA outside of *jbs* (C-90 in Figure 5). Unspecific DNA binding was also observed in the band shift experiments and is possibly due to our *in vitro* conditions which do not reflect the true *in vivo* situation. Weak DNA binding and low specificity have also been observed for TraJ_F_ binding to the FP_Y_ promoter; in addition, the authors of that study suggested a cooperative binding mode without a direct protein-protein interaction (Lu et al., 2018). Our findings that ArcA-P binding to R1P_Y_ DNA somehow enhanced TraJ_R__1_ binding to *jbs* containing DNA is consistent with this notion. Although we observed no direct protein-protein interaction between TraJ_R__1_ and ArcA (data not shown), cooperativity between ArcA and TraJR1 could also be an important aspect of R1P_Y_ activation. Furthermore, results of several experiments suggest a direct interaction of TraJ with RNA polymerase (G. Koraimann, unpublished observations). Such a complex may strongly enhance affinity and sequence specificity. Investigations to better understand the interaction and complex formation between TraJ and the RNA polymerase of *E. coli* are currently ongoing in our laboratory.

In stark contrast to the *in vitro* observations are the results of R1P_Y_ promoter mutations in *jbs* and the effects of these mutations on the activation potential by TraJ_R__1_ or TraJ_F_. The sequence identity between these two TraJ variants is only 18%—explaining the observed specificity of these activators, which means that TraJ_R__1_ activates only the R1_PY_ promoter whereas TraJ_F_ only functions to activate the FP_Y_ promoter containing the cognate *jbs* site (Wagner et al., 2013; Lu et al., 2014). Based on TraJ sequence variations within the MOB_F__12_A family of F-like plasmids, nine subgroups have been recently proposed, presumably resulting in a subgroup-specific activation of the P_Y_ promoter (Koraimann, 2018). Two groups that share a rather high identity in the TraJ protein sequence (73%) are the R1 subgroup and the pSLT subgroup. As we demonstrate here, TraJ_R__1_ and TraJ_pSLT_ are exchangeable. Furthermore, there are only a few bases that differ in the respective promoter sequences (Figure 1). Exchanges of these bases, mostly outside of *jbs* did not affect silencing nor activation by TraJ_R__1_ or TraJ_pSLT_ (Supplementary Figure S1). However, when *jbs* in R1P_Y_ was mutated in only three positions of the inverted repeat sequence, we immediately observed a loss of activation by TraJ_R__1_ indicating that recognition by TraJ *in vivo* is extremely specific. After a series of mutations toward the *jbs* sequence in FP_Y_, we finally observed a specificity switch, as the promoter was activated by TraJ_F_ and not by TraJ_R__1_. This result shows that the sole determinant for recognition by TraJ resides in the identified *jbs* sequence.

## Data Availability Statement

The datasets generated for this study are available on request to the corresponding author.

## Author Contributions

KB, DS, ST, TH, AH, and GR performed and analyzed the experiments. GK designed, evaluated, and analyzed the data and wrote the manuscript.

## Conflict of Interest

The authors declare that the research was conducted in the absence of any commercial or financial relationships that could be construed as a potential conflict of interest.

## Footnotes

1 https://openwetware.org/wiki/?title=Knight:Beta-galactosidase_assay/96_well_format&oldid=194997
