# Supplementary figures and images for: Regulation of R1 Plasmid Transfer by H-NS, ArcA, TraJ, and DNA Sequence Elements
Source: Front Microbiol. 2020 Jun 11;11:1254. doi: 10.3389/fmicb.2020.01254 (PMC7303359; doi:10.3389/fmicb.2020.01254)

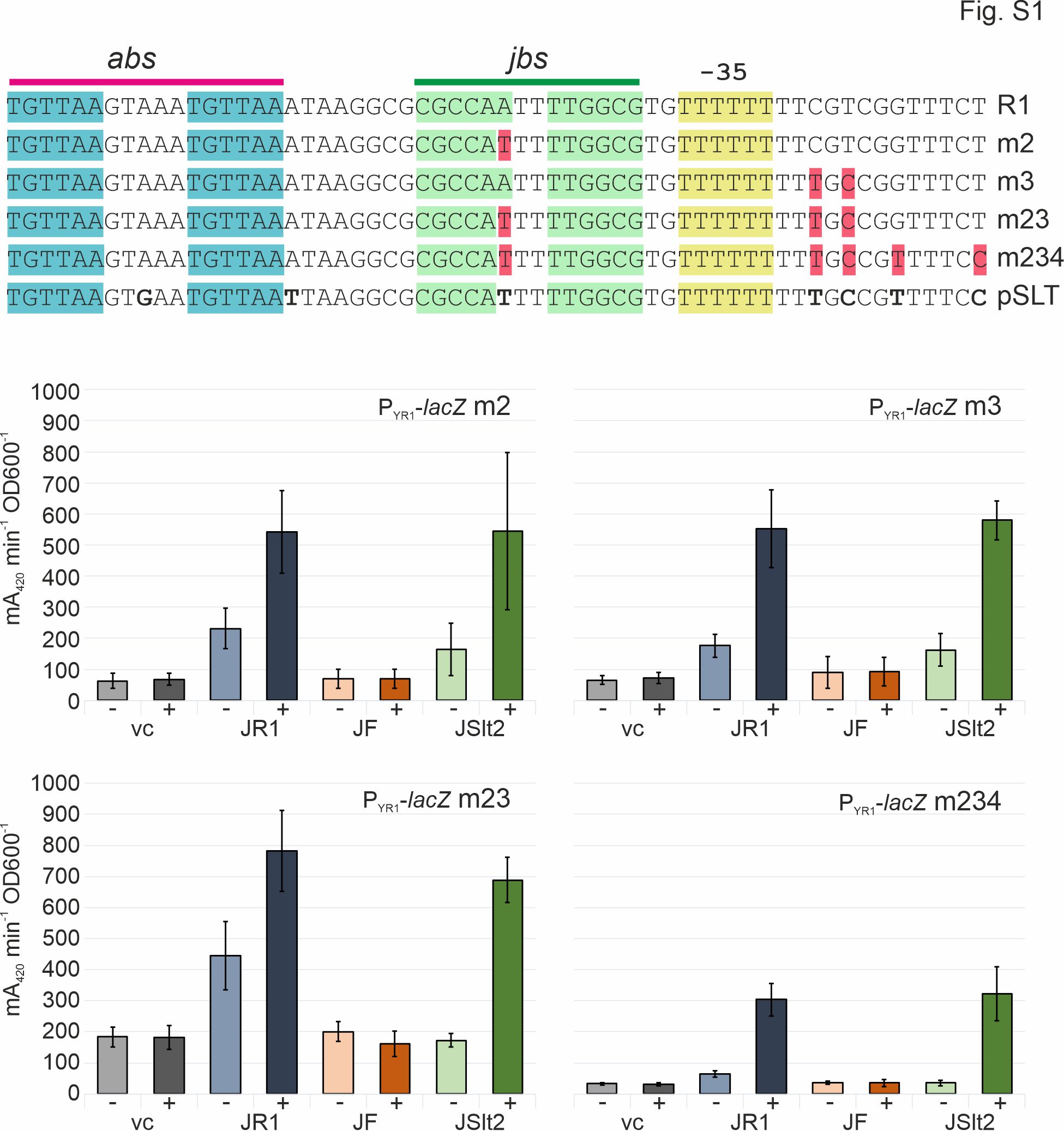

Supplement: Supplementary file 1 [file Image_1.JPEG]
