# Supplementary material for: Regulation of R1 Plasmid Transfer by H-NS, ArcA, TraJ, and DNA Sequence Elements
Source: Front Microbiol. 2020 Jun 11;11:1254. doi: 10.3389/fmicb.2020.01254 (PMC7303359; doi:10.3389/fmicb.2020.01254)
Supplement: Supplementary file 2 [file Table_1.docx]

# Supplementary Information

**Regulation of F plasmid transfer by proteins and DNA sequence elements.**

Karin Bischof, Doris Schiffer, Sarah Trunk, Thomas Höfler, Anja Hopfer*,* Gerald Rechberger, and Günther Koraimann

# Supporting Materials and Methods

## TraJ expression construct pSD1002

For construction of pSD1002, the expression vector of the His-MBP-TraJ fusion protein the *malE* gene (encoding for MBP) was amplified by PCR from plasmid pMAL-c2x with primers malENHis_fw and malECTEV_rev introducing an N-terminal His-tag and a C-terminal TEV protease cleavage site. *traJ* was PCR amplified from *E. coli* J5 harboring plasmid R1 with the primers traJNTEV_fw and traJ-C_rev introducing a N-terminal TEV protease cleavage site. The *malE* and *traJ* fragments were used for overlap extension PCR to generate the His-*malE*-TEV-*traJ* gene fusion which was first cloned into pUC119 using the *Eco*RI/*Xba*I restrictions sites and clones were selected on LB-glu agar plates containing 100 µg/mL ampicillin. The *traJ* sequence in the resulting plasmid pSD1001 was verified by DNA-sequencing. Subsequently, the *Eco*RI/*Xba*I fragment was subcloned into pGZ119EH, resulting in pSD1002. The first transformants after subcloning were grown at room temperature since colony formation at 37°C did not occur. The functionality of the fusion protein was confirmed by its ability to activate conjugative DNA transfer of the repressed R1 derivative pAR183 (Reisner et al. 2006) in a mating assay.

## H-NS expression construct pSThns

The *hns* gene was amplified by PCR from *E. coli* MG1655 using the primers hns_nde_fw and hns_eco_rev. The purified 434 bp *hns* fragment and the expression vector pET28a(+) were digested with restriction endonucleases NdeI and EcoRI. Vector and insert fragments were subsequently ligated and electro-transformed into *E. coli* XL-1 Blue cells. The resulting pSThns plasmid (5744 bp) was isolated from transformants, verified by DNA sequencing and used to transform *E. coli* BL21-(DE3) cells for His_6_-H-NS protein production.

## Cultures for protein production

Overnight cultures of *E. coli* BL21 harboring the His-MBP-TraJ expression plasmid pSD1002 or pSD1002^I187T^ were inoculated to an initial OD_600_ of 0.1 in 500 mL LB medium, supplemented with 20 µg/mL chloramphenicol in a 2 L Erlenmeyer flask and incubated at 37°C. *E. coli* BL21(DE3) containing pSThns was similarly grown in a 100 ml culture containing 40 µg/ml kanamycin. Cultures were grown to an OD_600_ of 0.5 to 0.7 and expression was induced by addition of 1 mM IPTG (final concentration) for 2 h. Cells were then harvested by centrifugation using Beckman Coulter Avanti(R) J-26XP centrifuge with the rotor JA 10 for 5 minutes and 5,000 rpm at 4°C. The pellet was resuspended in 15 mL 1x PBS buffer (0.5 M NaCl, 20 mM sodium phosphate buffer, pH 7.4) and transferred to a 15 mL Sarstedt tube. The centrifugation was repeated as described above using rotor JA 25.50. This washing step was repeated twice, the resulting pellet was either stored at -20°C or directly used for bacterial cell disruption.

## TraJ purification

Cell pellets were suspended in chromatography buffer (20 mM sodium phosphate buffer pH 7.4, 150 mM NaCl) containing 5 mM Tris[2-carboxyethyl]phosphine (TCEP) and Complete protease inhibitor cocktail tablets (Roche) and lysed using a French press. The cell lysates were cleared by centrifugation (45 min, 30000 x g, 4°C) and the soluble fraction applied to an amylose resin (New England Biolabs) column to purify His-MBP-TraJ. The column was washed with chromatography buffer and subsequently with chromatography buffer without protease inhibitor. His-MBP-TraJ was eluted with 10 mM maltose in chromatography buffer. Protein concentrations were determined according to Bradford using the Bio-Rad Protein Assay (Bio-Rad). Fractions containing the fusion protein as identified by SDS-PAGE and Western blotting were pooled and digested with 1 µg His-tagged TEV protease per 1 mg His-MBP-TraJ over night at room temperature. Insoluble protein was removed by centrifugation (15 min, 30000 x g, 25°C) and the soluble fraction was supplemented with 20 mM imidazole and used for subtractive affinity purification with a 1 mL HisTrap™ HP Column (GE Healthcare) to remove His-MBP and His-TEV protease. Flow through fractions containing TraJ were collected.

## His_6_-H-NS purification

Cell pellets were resuspended in 3 ml chromatography buffer with TCEP and protease inhibitor (see above) and cells were disrupted by sonication using a Branson Sonifier 250. Samples were sonicated five times for 15 sec and placed on ice between the sonication steps. Cell debris was removed by centrifugation for 10 min at 4°C and 12,000 g. His_6_-HNS was bound to a 1 mL HisTrap™ HP Column (GE Healthcare) in the presence of 20 mM imidazole to reduce unspecific binding. After several washing steps with buffer containing 60 mM or 200 mM imidazole His_6_-H-NS eluted with chromatography buffer containing 500 mM imidazole.

**Protein concentration, buffer exchange and storage**

After elution from affinity columns buffer exchange and protein concentration was performed using Amicon Ultra-15 centrifugal filter units (10,000 NMWL, Millipore). Samples were pipetted into the ultrafiltration units and centrifuged with 7,500 g at 4°C to reduce the volume to approximately 400 µL. Next, 1 ml of storage buffer was added, and samples were centrifuged as above. This step was repeated twice. Finally, protein samples in storage buffer were stored in 50 µl aliquots at -80°. Storage buffer for TraJ: 50% glycerol, 10 mM Tris-Cl (pH 7.9), 0.5 M NaCl, 0.1 mM EDTA (pH 8.0), 5 mM TCEP. Storage buffer for His_6_-H-NS: 50% glycerol, 10 mM Tris-Cl (pH 7.9), 0.3 M KCl, 0.1 mM EDTA (pH 8.0), 0.1 mM DTT.

## β-galactosidase assays in 96 well plates

Pre-cultures in LB medium (supplemented with supplemented with chloramphenicol and ampicilliin) were prepared from inoculating aliquots of frozen glycerol stocks. Precultures grown over night were used to inoculate 10 ml prewarmed M9 minimal salt medium medium (without and with IPTG, 0.1 mM final concentration) to an OD_600_ of 0.1 in 50 ml screw-cap centrifugation tubes and further incubated for 90 min at 37°C. After cooling on ice and OD_600_ determination cells were harvested by centrifugation (5 min, 13000 rpm, 4°C). The cell pellets were resuspended in PBS to an OD_600_ of 0.5 per ml. Two pellets of 0.4 OD_600_ units (for β-galactosidase assays) and one pellet of 0.5 OD_600_ units (for SDS PAGE and Western Blotting) were harvested and kept at -20°C until further use. The frozen cell pellets (0.4 OD_600_ units) were resuspended in 400 μl of permeabilization solution (100 mM Na_2_HPO_4_, 20 mM KCl, 2 mM MgSO_4_, 0.06 % CTAB, 0.04 % sodium deoxycholate, 0.54 % TCEP). The resuspended cells were transferred to two wells of a 96-well-plate (100 μl each). After incubation at room temperature for 80 minutes, 25 μl corresponding to 0.025 OD_600_ units were mixed with 150 µL of a substrate solution (60 mM Na_2_HPO_4_, 40 mM NaH_2_PO_4_, 1 mg/mL ONPG, 0.27 % TCEP) in a second 96 well plate. β-galactosidase activity was determined by measuring the increase of mOD_420_ (milli-OD_420_: Absorption at 420 nm x 1000) per min in a Spectrostar Nano (BMG Labtech) microplate reader for 30 min with 90 sec time intervals. The obtained kinetic curves were analyzed using MARS data analysis software and corrected by subtracting OD_550_ values at each time point. The slope was calculated from a linear regression fit of each OD_550_ corrected curve. β-galactosidase activities reflect promoter activation and are expressed as mOD_420_ min^-1^ OD_600_^-1^. Further data analysis including statistics was performed using Microsoft Excel.

## Table S1: *E. coli* strains and plasmids

| ***E. coli* strains** | **Genotype or description** | **Source and/or reference** |
| --- | --- | --- |
| BL21 | *dcm ompT lon hsdS*(r_B_- m_B_-) *gal* [*malB^+^*]_K-12_(λ^S^) | Stratagene / (Studier and Moffatt 1986) |
| BL21(DE3) | BL21, λ phage integrated with gene 1 of bacteriophage T7 encoding T7 RNA polymerase under *lac* promoter control | (Studier and Moffatt 1986) |
| XL-1 Blue | *endA1 gyrA96(nal^R^) thi-1 recA1 relA1 lac glnV44 F'[ ::*Tn*10 proAB^+^ lacI^q^ Δ(lacZ)M15] hsdR17(r_K_^-^ m_K_^+^)* | Stratagene |
| MC4100 | *[araD139]_B/r_, Δ (argF-lac)169, flhD5301, Δ (fruK-yeiR)725(fruA25), relA1, rpsL150(strR), rbsR22, Δ (fimB-fimE)632(::IS*1*), deoC1* | Strain collection |
| **Plasmids** |  |  |
| pUC119 | High copy number cloning vector, pMB1 *ori*, Ap^R^ | (Vieira and Messing 1987) |
| pMal-c2X | Expression vector, pMB1 *ori*, *malE*, Ap^R^ | New England Biolabs |
| pGZ119EH | Low copy number expression vector, ColD *ori*, *lacI*^q^, P_tac_, T_rrnB_, Cm^R^ | (Lessl et al. 1992) |
| pET28a(+) | Expression vector, 5369 bp, pMB1 *ori*, T7 promoter, *lacI*^q^ , Kan^R^ | Novagen |
| pSD1001 | His-MBP-tagged *traJ* from plasmid R1 cloned into the *EcoR*I/ *Xba*I site of pUC119, Ap^R^ | This study |
| pSD1002 | His-MBP-tagged *traJ* subcloned from pSD1001 into *EcoR*I/*Xba*I sites of pGZ119EH, Cm^R^ | This study |
| pSThns | pET28a(+) expression vector with *hns* gene from E. coli MG1655, 5744 bp, Kan^R^ | This study |
| pGZNSO2 | ﻿pGZ119EH with one additional symmetrical *lac* operator sequence for low inducible expression or *traJ* orthologues, Cm^R^ | (Wagner et al. 2013) |
| pJR1 | N-terminally FLAG-tagged *traJ* from plasmid R1 with a *finP* promoter mutation cloned into pGZNSO2 | (Wagner et al. 2013) |
| pJF | N-terminally FLAG-tagged *traJ* from plasmid F with a *finP* promoter mutation cloned into pGZNSO2 | (Wagner et al. 2013) |
| pJSLT2 | N-terminally FLAG-tagged *traJ* from plasmid pSLT with a *finP* promoter mutation cloned into pGZNSO2 – recloned because the original pJSLT had a deletion in the lac operator sequence. | This study |
| pRSYZ4 | MunI/XhoII fragment with P_Y_ promoter of plasmid R1 cloned into the promoter test vector pRS414, Amp^R^ |  |
| pRSYZ4_mx_ | Derivatives of pRSYZ4 with mutations in R1P_Y_: m1, m4, m41, m42, m43, m6, m7, m61, m42a, m42a6, m2, m3, m23, m234 | This study |

## Table S2: Oligonucleotides used in this study

| **Oligonucleotide** | **Sequence** | |
| --- | --- | --- |
| **PCR cloning primers** | | |
| malENHis_fw | | TAGAATTCATGGGTCACCATCACCACCATCACAAAACTGAAGAAGGTAAACTGG |
| malECTEV_rev | | ACCCTGGAAGTACAGGTTCTCGAGGTTGTTGTTATTGTTATT |
| traJNTEV_fw | | GAGAACCTGTACTTCCAGGGTTGTGCGCTGGACCGTAGA |
| traJ-C_rev | | CGTCTAGATTATTACTTAACACCATAAAATTCACG |
| **Primers for EMSA, DNase I footprinting, promoter mapping** | | |
| PYTransfw | | TGGTCACGCAACAAT TGCC |
| PYTransrev1 | | TGTTCTGCTTATCCCGCCTC |
| pTG-NF4-fw | | CGGCTCGTATAATGTGTGGAATTG |
| pgzrev508 | | GCTACGGCGTTTCACTTCTG |
| F_Py_fw | | GCTGCTCATGTTCGTCATAAAG |
| F_Py_rev | | TAACACCTCCCGCTGTTTATCT |
| GroEL_Atum_fw | | ATGAACCCGATGGACCTCAAG |
| GroEL_Atum_rev | | TGAACGACGGCTTCGAGAAC |

# Supporting Results

## Fig. S1

**Fig. S1:** Determination of important sequence elements in the R1P_Y_ promoter for activation by TraJ_R1_. Successively introduced mutations switch recognition specificity to TraJ_pSLT_. **A**. Sequences showing sequentially created site specific mutations in R1P_Y_. Top line shows the R1P_Y_ wt sequence with the *abs* and *jbs* sites indicated above. Bottom line shows pSLTP_Y_ wt sequence with bases that differ from R1P_Y_ in bold letters. **B**. Results of β-galactosidase assays with bacterial cells harboring a R1P_Y_-*lacZ* promoter-test plasmid and a second compatible plasmid IPTG-inducible for expression of *traJ* from plasmids R1 (JR1), F (JF) or pSLT (JSlt2); vc: vector control (no *traJ*). IPTG (inductor): + (added), - (not added). Mean values and standard deviations were calculated from at least three independently carried out experiments with two technical replicates for each experiment. Differences between JR1 (-/+ IPTG) and JSLT2 (-/+ IPTG) were highly significant as were the differences between TraJ expressing (IPTG +) cells (R1 or pSLT) versus vector control (P-values according to paired t-test were <0.001).

# Supporting References

Lessl, M, D Balzer, R Lurz, V L Waters, D G Guiney, and E Lanka. 1992. “Dissection of IncP Conjugative Plasmid Transfer: Definition of the Transfer Region Tra2 by Mobilization of the Tra1 Region in Trans.” *Journal of Bacteriology* 174 (8): 2493–2500. https://doi.org/10.1128/JB.174.8.2493-2500.1992.

Reisner, Andreas, Brigitte M Höller, Søren Molin, and Ellen L Zechner. 2006. “Synergistic Effects in Mixed Escherichia Coli Biofilms: Conjugative Plasmid Transfer Drives Biofilm Expansion.” *Journal of Bacteriology* 188 (10): 3582–88. https://doi.org/10.1128/JB.188.10.3582-3588.2006.

Studier, F W, and B A Moffatt. 1986. “Use of Bacteriophage T7 RNA Polymerase to Direct Selective High-Level Expression of Cloned Genes.” *J Mol Biol* 189 (1): 113–30.

Vieira, J, and J Messing. 1987. “Production of Single-Stranded Plasmid DNA.” *Methods Enzymol* 153: 3–11.

Wagner, Maria Anna, Karin Bischof, Dominiki Kati, and Günther Koraimann. 2013. “Silencing and Activating Type IV Secretion Genes of the F-like Conjugative Resistance Plasmid R1.” *Microbiology* 159 (Pt_12): 2481–91. https://doi.org/10.1099/mic.0.071738-0.
